# Fasting remnant lipoproteins can predict postprandial hyperlipidemia

**DOI:** 10.1186/1476-511X-11-146

**Published:** 2012-10-31

**Authors:** Tomoki Nagata, Daisuke Sugiyama, Takako Kise, Satomi Tsuji, Hideo Ohira, Itsuko Sato, Mari Yamamoto, Hitomi Kohsaka, Seiji Kawano, Shizuya Yamashita, Yuichi Ishikawa, Yoshio Fujioka

**Affiliations:** 1 Division of Clinical Nutrition, Faculty of Nutrition, Kobe Gakuin University, 518 Arise, Ikawadani-cho, Nishi-ku, Kobe, 651-2180, Japan; 2 Section of Evidence-based Laboratory Medicine, Division of Clinical Pathology and Immunology, Department of Internal Medicine Related, Kobe University Graduate School of Medicine, Kobe, Japan; 3 Department of Clinical Laboratory, Kobe University Hospital, Kobe, Japan; 4 Department of Cardiovascular Medicine, Osaka University Graduate School of Medicine, Osaka, Japan; 5 Department of Internal Medicine, Kakogawa West City Hospital, Kakogawa, Japan; 6 Division of Clinical Laboratory, Shimane Prefectural Central Hospital, Shimane, Japan; 7 Department of Preventive Medicine and Public Health, School of Medicine, Keio University, Tokyo, Japan; 8 Division of Nutrition Guidance, Kakogawa East City Hospital, Kakogawa, Japan

**Keywords:** Remnant lipoprotein, Postprandial hyperlipidemia, Triglyceride, Apolipoprotein B, Adiponectin.

## Abstract

**Background:**

Hypertriglyceridemia and postprandial hyperlipidemia is thought to play an important role in atherosclerosis, but to select patients at high-risk for cardiovascular diseases is difficult with triglycerides (TG) alone in these patients.

**Methods:**

To predict postprandial hyperlipidemia without inconvenient test meal loading, we examined lipid concentrations before and after test meal loading and fasting adiponectin, and investigated which of these other than TG were significant during the fasting period in 45 healthy individuals (men: women, 26:19).

**Results:**

TG, remnant-like particle-cholesterol and -triglyceride (RemL-C, RLP-C, and RLP-TG), and TG/apolipoprotein(apo)B were significantly elevated after loading and fasting values significantly and positively correlated with incremental area under the curve (iAUC) (r=0.80, r=0.79, r=0.63, r=0.58, r=0.54; p<0.0001). Fasting adiponectin positively correlated with fasting high-density lipoprotein-cholesterol (r=0.43, p<0.005) and apoA-I (r=0.34, p<0.05), and negatively correlated with iAUC of TG, RemL-C, RLP-C, RLP-TG, and TG/apoB (r=−0.37, r=−0.41, r=−0.37, r=−0.36, r=−0.37; p<0.05). We constructed the model of multivariable linear regression analysis without fasting TG. In the sex-, BMI-, age-, and waist circumference-adjusted analysis of postprandial TG elevation 2 h after test meal loading in all participants, RemL-C, RLP-C, RLP-TG, and TG/apoB were significant factors, but adiponectin was not.

**Conclusion:**

Fasting triglyceride-rich lipoprotein-related values, especially RemL-C, RLP-C, RLP-TG, and TG/apoB are useful predictors of postprandial hyperlipidemia in young healthy individuals. Although fasting adiponectin concentration correlated with the iAUCs for TG, RemL-C, RLP-C, RLP-TG, and TG/apoB, it was not a significant predictor of postprandial hyperlipidemia in multivariable linear regression analysis.

## Background

Epidemiological studies have recently shown that hypertriglyceridemia is associated with atherosclerosis, but the independence of the serum triglycerides (TG) concentration as a causal factor in promoting cardiovascular diseases (CVD) remains debatable and to select patients at high-risk for CVD is difficult with TG alone [1-3]. Individuals with mild hypertriglyceridemia without other metabolic disorders or severe hypertriglyceridemia such as primary chylomicronemia rarely have CVD.

Postprandial hyperlipidemia is thought to play an important role in atherosclerosis, and concentrations of non-fasting TG are superior to those of fasting TG for predicting CVD [4-7]. Many studies have revealed that triglyceride-rich lipoproteins (TRL), especially chylomicron and very-low-density lipoprotein (VLDL) remnants, are atherogenic and that delayed removal of chylomicron remnants from the bloodstream induces postprandial hyperlipidemia [8-10]. However, screening large numbers of individuals using fat loading tests is inconvenient and neither a definition nor a standard method for predicting postprandial hyperlipidemia besides postprandial TG elevation has been established. These circumstances present a challenge in terms of how to distinguish patients at high-risk of CVD based on fasting blood samples. We reported that fasting serum concentrations of remnant lipoproteins and apolipoprotein B-48 (apoB-48) besides TG might indicate postprandial hyperlipidemia even among normolipidemic individuals[11].

Adipocytes secrete adiponectin, which is a 224-amino-acid plasma protein [12,13]. Serum adiponectin concentrations are paradoxically reduced in individuals with a large visceral fat mass, and adiponectin plays a significant role in glucose and lipid metabolism [13-15]. However, few studies have examined the relationship between serum adiponectin concentrations and postprandial hyperlipidemia. Rubin et al. demonstrated that postprandial TG concentrations correlate with fasting adiponectin concentrations in 45 - 65-year-old individuals, including those with metabolic syndrome [16]. Maruyama et al. reported that serum concentrations of high molecular weight (HMW) adiponectin are associated with those of TG and remnant-like particle-triglyceride (RLP-TG) in individuals with type 1 diabetes before and after test meal loading [17].

Thus, the significance of adiponectin in postprandial hyperlipidemia needs to be clarified. Here, we used multivariable linear regression analysis to identify which factors among remnant lipoproteins, adiponectin and other particles during fasting are significant for predicting postprandial hyperlipidemia.

## Methods

### Participants and physical examination

We recruited 45 healthy individuals (men/women, 26/19) who had never been treated for diseases or taken drugs for at least 3 months prior to the study. We used data from 24 participants that we had previously analyzed [11] and we added another 21 individuals for the present study. All of the recruits provided written, informed consent to participate in the study. The institutional ethics committee of Kobe Gakuin University (HEB080701-1) approved the study protocol, which proceeded according to the Declaration of Helsinki. We measured the waist circumference and computed the body mass index (BMI) of all participants by dividing body weight by the square of the height (kg/m^2^).

### Study protocol

The study proceeded as previously described [11]. Test meal A was developed by the Japanese Diabetes Society to assess both postprandial hyperglycemia and hyperlipidemia. This meal consisted of cream of chicken soup, a biscuit and custard pudding. The total 450 kcal of energy was derived from 57.6g of carbohydrate (51.4% of the energy balance), 17.2g of protein (15.3%), and 16.6 g of fat (33.3%), which is a slightly higher ratio of fat than that found in a typical Japanese breakfast (20% - 25%). Blood samples were obtained between 9 and 10 AM after 12 h of fasting and at 1, 2, 4, 6 and 8 h after test meal loading.

### Blood analysis

Serum concentrations of HMW adiponectin and plasma-oxidized low-density lipoproteins (LDL) were determined using sandwich enzyme-linked immunosorbent assays (ELISA) (Fujirebio, Tokyo, Japan and Kyowa Medex, Tokyo, Japan, respectively). Serum concentrations of TG, total cholesterol, high-density lipoprotein-cholesterol (HDL-C) were determined enzymatically (Kyowa Medex, Tokyo, Japan). For low-density lipoprotein-cholesterol (LDL-C), we used the commercial kit by the selective solubilization method (Determiner L LDL-C, Kyowa Medex) because it is impossible to use Friedewald’s formula in the postprandial cases. Serum concentrations of apoA-I, apoA-II, apoB, apoC-II, apoC-III, and apoE were measured using turbidimetric immunoassays (Nittobo, Tokyo, Japan). Serum small dense LDL-cholesterol (sd-LDL-C) concentrations were measured using a precipitation method (Denka Seiken, Tokyo, Japan) [18]; serum concentrations of remnant-like particle-cholesterol (RLP-C) and RLP-TG were determined using immune adsorption (JIMRO II, Otsuka Pharmaceutical, Tokyo, Japan) [19]; serum remnant lipoprotein cholesterol (RemL-C) concentrations were measured using a homogeneous assay (MetaboLead RemL-C, Kyowa Medex) [20]. Serum high-sensitivity C-reactive protein (hs-CRP) concentrations were determined using nephelometry (Dade Behring, Deerfield, IL, USA). Plasma glucose concentrations were determined enzymatically. Glycosylated hemoglobin A1c (HbA1c) values were determined using HPLC. Serum insulin concentrations were measured using an enzyme immunoassay. Non-HDL-C concentrations were computed by subtracting the value of HDL-C from that of TC.

### Statistical analysis

Values are expressed as means ± SD. Data below the threshold of RLP-C (< 2.0mg/dl) or RLP-TG (< 15mg/dL) were treated as 2.0 or 15 mg/dL, respectively. TG, RemL-C, RLP-C, RLP-TG, TG/apoB, and adiponectin were transformed into logarithmic values. The statistical significance of the data was evaluated using Welch’s *t*-test or repeated ANOVA with Dunnett’s test. Postprandial changes in values of TG, RemL-C, RLP-C, RLP-TG, or TG/apoB were quantified by calculating the incremental area under the curve (iAUC), which was estimated as the difference between the area defined below the baseline concentration and the area under the curve in each factor or parameter from 1 h to 8 h after test meal loading.

Correlations between adiponectin and lipids as well as other factors were calculated using the formula for Pearson’s correlation coefficient.

Independent predictors of TG at 2 h after test meal loading were identified by multivariable linear regression analysis adjusted with sex, BMI, age, waist circumference. Candidate predictors (RemL-C, RLP-C, RLP-TG, TG/apoB, and adiponectin) were analyzed separately because of correlation among candidate predictors. The purpose of our study was to elucidate the factors that predict postprandial hyperlipidemia besides TG, because it is difficult to select high-risk patients with TG alone. Therefore, we did not investigate fasting TG as candidate predictor in regression model. Data were statistically analyzed using SPSS Statistics 17.0 (IBM, Somers, NY, USA) and R version 2.12.1 (R Foundation for Statistical Computing, Vienna, Austria). Two-tailed values of *p* < 0.05 were considered statistically significant.

## Results

### Characteristics of subjects

Table 1 shows the characteristics of the 26 men and 19 women participants, except for LPL mass (total 21; 15 men and 6 women). Although all factors were within normal limits, the values of height, body weight, waist circumference, and hs-CRP concentration were higher in men than in women. On the other hand, adiponectin concentration was higher in women than in men.

**Table 1:** Baseline characteristics of study participants

		**Total**	**Men**	**Women**
Sex	(n)	45	26	19
Age	(years)	21.5 ± 1.3	21.4 ± 1.5	21.7 ± 0.9
Height	(m)	1.66 ± 0.08	1.70 ± 0.06	1.61 ± 0.07*
Body weight	(kg)	58.1 ± 8.8	61.8 ± 9.3	53.1 ± 4.9^†^
Waist circumference	(cm)	73.8 ± 7.8	73.5 ± 7.5	67.9 ± 5.44^‡^
BMI	(kg/m^2^)	21.0 ± 2.3	21.4 ± 2.8	20.5 ± 1.4
HbA1c	(%)	4.9 ± 0.2	4.91 ± 0.23	4.91 ± 0.25
Hs-CRP	(mg/dL)	0.04 ± 0.02	0.05 ± 0.02	0.03 ± 0.02^§^
Adiponectin	(μg/mL)	5.53 ± 3.13	4.51 ± 2.38	6.93 ± 3.54^‡^
LPL mass	(ng/mL)	44.1 ± 12.7	42.3 ± 12.1	48.8 ± 15.3

### Fasting and postprandial concentrations of lipids and their parameters

Tables 2A (all participants) shows the changes of lipid, glucose, and insulin concentrations before and after test meal loading. Since some metabolic factors in Table 1 differed between men and women, we also compared these data in Table 2B (men) and C (women).

**Table 2:** Lipid and glucose parameters for before and 1 – 8 h after loading with test meal

**A. All participants**	**Elapsed time (h)**						
		**0**	**1**	**2**	**4**	**6**	**8**
TC	(mg/dL)	182.4 ± 32.8	180.2 ± 32.8	178.2 ± 31.3	180.4 ± 30.1	184.0 ± 31.6	188.2 ± 32.5
LDL-C	(mg/dL)	98.5 ± 26.1	96.3 ± 25.5	95.6 ± 25.5	96.7 ± 24.7	99.8 ± 25.6	102.1 ± 26.2
HDL-C	(mg/dL)	70.1 ± 15.4	68.3 ± 15.4	66.2 ± 14.1	68.3 ± 15.0	70.2 ± 15.7	72.0 ± 16.0
Sd-LDL-C	(mg/dL)	22.4 ± 12.1	19.8 ± 8.8	18.8 ± 8.5	18.6 ± 7.1	18.6 ± 7.1	19.0 ± 7.0
OxLDL	(U/mL)	7.38 ± 4.96	7.04 ± 4.98	7.25 ± 5.74	7.41 ± 5.53	7.71 ± 5.41	8.38 ± 5.46
TG	(mg/dL)	69.8 ± 31.2	92.1 ± 41.0*	103.9 ± 51.4^†^	72.9 ± 40.4	70.7 ± 34.3	53.9 ± 23.8
Non-HDL-C	(mg/dL)	112.3 ± 30.1	112.0 ± 29.9	112.0 ± 29.3	112.0 ± 28.4	113.8 ± 28.7	116.3 ± 29.4
RemL-C	(mg/dL)	3.51 ± 1.84	4.35 ± 2.11	4.29 ± 2.46	3.90 ± 2.38	3.22 ± 1.61	2.98 ± 1.40
RLP-C	(mg/dL)	3.20 ± 1.30	4.22 ± 1.91*	5.01 ± 2.42^‡^	3.88 ± 1.94	3.03 ± 1.02	2.82 ± 0.88
RLP-TG	(mg/dL)	16.3 ± 4.0	25.5 ± 14.4^†^	32.6 ± 21.8^‡^	21.1 ± 12.3	15.8 ± 3.4	15.3 ± 2.0
ApoA-I	(mg/dL)	161.2 ± 26.6	159.8 ± 27.3	158.7 ± 25.2	160.3 ± 25.2	163.3 ± 26.4	165.7 ± 27.0
ApoA-II	(mg/dL)	37.6 ± 6.0	37.3 ± 6.2	36.8 ± 6.0	37.4 ± 5.9	37.7 ± 5.9	38.4 ± 6.1
ApoB	(mg/dL)	66.6 ± 15.3	65.7 ± 15.1	65.0 ± 14.5	66.2 ± 14.5	67.9 ± 14.8	69.3 ± 15.2
ApoC-II	(mg/dL)	3.2 ± 1.2	3.3 ± 1.2	3.3 ± 1.2	3.3 ± 1.2	3.3 ± 1.1	3.3 ± 1.1
ApoC-III	(mg/dL)	9.5 ± 2.4	9.8 ± 2.5	9.4 ± 2.3	9.2 ± 2.2	9.0 ± 2.1	9.0 ± 2.2
ApoE	(mg/dL)	4.3 ± 1.1	4.3 ± 1.1	4.3 ± 1.0	4.2 ± 1.1	4.1 ± 1.0	4.2 ± 1.1
Non-HDL-C/HDL-C		1.70 ± 0.73	1.75 ± 0.75	1.80 ± 0.75	1.75 ± 0.76	1.73 ± 0.74	1.72 ± 0.73
LDL-C/HDL-C		1.50 ± 0.60	1.50 ± 0.61	1.52 ± 0.61	1.50 ± 0.61	1.51 ± 0.60	1.50 ± 0.60
ApoB/apoA-I		0.43 ± 0.13	0.42 ± 0.13	0.42 ± 0.13	0.43 ± 0.13	0.43 ± 0.13	0.43 ± 0.13
Non-HDL-C/apo B		1.67 ± 0.10	1.69 ± 0.10	1.71 ± 0.11	1.68 ± 0.10	1.67 ± 0.10	1.67 ± 0.10
TG/apoB		1.06 ± 0.44	1.41 ± 0.56^§^	1.61 ± 0.71^‡^	1.25 ± 0.61	0.93 ± 0.40	0.79 ± 0.32
Plasma glucose		(mg/dL)	89.9 ± 5.7	96.9 ± 20.0^§^	87.5 ± 10.8	87.6 ± 4.9	88.1 ± 5.7
Insulin		(μU/mL)	5.6 ± 2.4	42.4 ± 24.6^‡^	18.5 ± 14.1^‡^	5.0 ± 2.2	4.2 ± 2.1
**B. Men**							
TC		(mg/dL)	183.1 ± 33.8	181.4 ± 34.5	180.0 ± 33.3	182.4 ± 32.5	185.0 ± 33.2
LDL-C		(mg/dL)	101.1 ± 28.2	99.0 ± 27.5	99.3 ± 27.7	99.8 ± 26.9	102.7 ± 27.8
HDL-C		(mg/dL)	66.3 ± 16.7	64.7 ± 17.4	62.5 ± 15.5	65.1 ± 17.2	66.5 ± 17.4
Sd-LDL-C		(mg/dL)	23.9 ± 11.0	22.2 ± 10.2	21.4 ± 9.5	20.4 ± 9.0	19.8 ± 8.0
OxLDL		(U/mL)	7.98 ± 5.48	7.91 ± 5.54	8.12 ± 6.19	8.32 ± 6.11	8.70 ± 6.22
TG		(mg/dL)	78.9 ± 36.0	106.3 ± 45.1	119.5 ± 58.9^†^	94.8 ± 51.2	68.6 ± 33.7
Non-HDL-C		(mg/dL)	116.8 ± 32.5	116.8 ± 32.2	117.5 ± 31.8	117.3 ± 31.1	118.5 ± 31.2
RemL-C		(mg/dL)	4.08 ± 2.13	4.99 ± 2.42	5.16 ± 2.81	4.69 ± 2.80	3.67 ± 1.91
RLP-C		(mg/dL)	3.62 ± 1.51	5.00 ± 2.03*	5.82 ± 2.76^‡^	4.53 ± 2.23	3.31 ± 1.13
RLP-TG		(mg/dL)	17.3 ± 5.1	30.0 ± 16.7^§^	37.8 ± 26.2^‡^	24.7 ± 15.2	16.4 ± 4.5
ApoA-I		(mg/dL)	155.7 ± 29.2	154.4 ± 29.9	154.3 ± 28.7	155.2 ± 29.0	157.9 ± 29.4
ApoA-II		(mg/dL)	33.8 ± 6.7	38.7 ± 6.8	38.4 ± 6.6	38.8 ± 6.5	39.1 ± 6.7
ApoB		(mg/dL)	68.8 ± 16.3	68.3 ± 16.3	67.5 ± 15.6	68.8 ± 15.8	70.5 ± 16.0
ApoC-II		(mg/dL)	3.5 ± 1.1	3.6 ± 1.1	3.6 ± 1.2	3.6 ± 1.2	3.6 ± 1.1
ApoC-III		(mg/dL)	9.7 ± 2.6	10.2 ± 2.8	9.8 ± 2.7	9.5 ± 2.5	9.2 ± 2.3
ApoE		(mg/dL)	4.1 ± 1.1	4.1 ± 1.1	4.2 ± 1.0	4.0 ± 1.1	3.9 ± 1.0
Non-HDL-C/HDL-C		1.90 ± 0.85	1.96 ± 0.88	2.02 ± 0.87	1.96 ± 0.89	1.94 ± 0.86	1.92 ± 0.86
LDL-C/HDL-C		1.64 ± 0.71	1.66 ± 0.72	1.70 ± 0.70	1.66 ± 0.72	1.67 ± 0.71	1.66 ± 0.71
ApoB/apoA-I		0.46 ± 0.15	0.46 ± 0.15	0.46 ± 0.15	0.46 ± 0.16	0.46 ± 0.15	0.46 ± 0.15
Non-HDL-C/apo B		1.69 ± 0.10	1.70 ± 0.11	1.73 ± 0.12	1.70 ± 0.11	1.67 ± 0.10	1.68 ± 0.11
TG/apoB		1.17 ± 0.52	1.58 ± 0.63	1.79 ± 0.84^†^	1.41 ± 0.73	1.00 ± 0.49	0.86 ± 0.39
Plasma glucose	(mg/dL)	91.1 ± 5.9	97.7 ± 19.9	85.7 ± 9.7	89.2 ± 4.6	88.7 ± 5.9	87.7 ± 5.9
Insulin	(μU/mL)	5.0 ± 2.1	33.6 ± 13.7^‡^	11.6 ± 7.4^†^	4.5 ± 2.0	3.8 ± 2.3	3.7 ± 1.8
**C. Women**							
TC	(mg/dL)	181.4 ± 32.3	178.6 ± 31.3	175.7 ± 29.1	177.6 ± 27.1	182.7 ± 30.0	186.5 ± 29.6
LDL-C	(mg/dL)	95.1 ± 23.3	92.6 ± 22.6	90.6 ± 21.8	92.5 ± 21.4	95.7 ± 22.2	97.7 ± 22.1
HDL-C	(mg/dL)	75.3 ± 12.0	73.2 ± 10.9	71.3 ± 10.3	72.8 ± 10.4	75.4 ± 11.6	77.0 ± 11.6
Sd-LDL-C	(mg/dL)	20.3 ± 13.6	16.5 ± 4.9	15.3 ± 5.3	16.1 ± 4.6	16.8 ± 5.5	16.7 ± 5.2
OxLDL	(U/mL)	6.55 ± 4.15	5.86 ± 3.91	6.06 ± 4.99	6.17 ± 4.48	6.37 ± 3.83	6.92 ± 4.10
TG	(mg/dL)	57.3 ± 16.8	72.7 ± 24.6	82.6 ± 28.3^‖^	63.7 ± 17.0	52.8 ± 12.4	45.2 ± 10.3
Non-HDL-C	(mg/dL)	106.1 ± 25.9	105.4 ± 25.8	104.4 ± 24.2	104.8 ± 23.2	107.3 ± 24.3	109.5 ± 23.9
RemL-C	(mg/dL)	2.73 ± 0.95	3.47 ± 1.16	3.11 ± 1.13	2.83 ± 0.95	2.60 ± 0.77	2.45 ± 0.69
RLP-C	(mg/dL)	2.62 ± 0.60	3.16 ± 1.06	3.90 ± 1.22^‡^	2.98 ± 0.88	2.66 ± 0.72	2.52 ± 0.54
RLP-TG	(mg/dL)	15	19.4 ± 7.0	25.5 ± 11.0^‡^	16.2 ± 2.1	15.0 ± 0.2	15
ApoA-I	(mg/dL)	168.7 ± 21.0	167.3 ± 21.8	164.8 ± 18.5	167.3 ± 17.3	170.7 ± 20.1	172.7 ± 20.3
ApoA-II	(mg/dL)	36.0 ± 4.6	35.4 ± 4.7	34.6 ± 4.4	35.3 ± 4.3	35.8 ± 4.1	36.5 ± 4.5
ApoB	(mg/dL)	63.7 ± 13.6	62.2 ± 12.9	61.5 ± 12.5	62.6 ± 12.2	64.4 ± 12.6	65.8 ± 12.4
ApoC-II	(mg/dL)	2.8 ± 1.1	2.9 ± 1.1	2.9 ± 1.1	2.9 ± 1.0	2.9 ± 1.0	2.9 ± 1.1
ApoC-III	(mg/dL)	9.1 ± 2.0	9.3 ± 2.0	8.9 ± 1.7	8.8 ± 1.6	8.6 ± 1.8	8.7 ± 1.8
ApoE	(mg/dL)	4.7 ± 1.0	4.6 ± 1.1	4.5 ± 1.1	4.5 ± 1.1	4.5 ± 1.1	4.6 ± 1.1
Non-HDL-C/HDL-C		1.43 ± 0.38	1.46 ± 0.40	1.48 ± 0.39	1.47 ± 0.40	1.45 ± 0.39	1.45 ± 0.37
LDL-C/HDL-C		1.28 ± 0.33	1.28 ± 0.33	1.29 ± 0.33	1.29 ± 0.34	1.29 ± 0.33	1.29 ± 0.32
ApoB/apoA-I		0.38 ± 0.08	0.37 ± 0.08	0.38 ± 0.08	0.38 ± 0.08	0.38 ± 0.08	0.38 ± 0.08
Non-HDL-C/apo B		1.66 ± 0.11	1.68 ± 0.09	1.69 ± 0.11	1.67 ± 0.08	1.66 ± 0.10	1.66 ± 0.10
TG/apoB		0.92 ± 0.26	1.17 ± 0.32^‡^	1.36 ± 0.40^†^	1.04 ± 0.29	0.84 ± 0.22	0.70 ± 0.17
Plasma glucose	(mg/dL)	88.3 ± 5.2	95.8 ± 20.6	89.9 ± 12.1	85.5 ± 5.0	87.1 ± 5.1	86.5 ± 5.7
Insulin	(μU/mL)	6.5 ± 2.6	54.1 ± 31.1^‡^	27.9 ± 15.7^‡^	5.6 ± 2.4	4.8 ± 1.8	3.9 ± 1.5

The concentrations of all parameters were within normal limits or at low ranges during the fasting period (time 0). Fasting values of TG*, RemL-C*, RLP-C^†^, RLP-TG^†^, non-HDL-C/HDL-C*, LDL-C/HDL-C* and apoB/apoA-I* were significantly greater in men than in women (*p < 0.05; ^†^*p* < 0.005, Welch’s *t*-test). Fasting RLP-TG concentrations in all women were undetectable (<15 mg/dL), and we set fasting RLP-TG concentration as 15 mg/dL in Table 2C. On the other hand, HDL-C concentrations were significantly lower in men than in women (*p* < 0.05, Welch’s *t*-test).

Serum concentrations of TG, RLP-C, RLP-TG, insulin and plasma glucose concentrations were significantly elevated after, compared with before loading in all participants (Table 2A). Serum RemL-C concentrations peaked at 1 h and were restored within 4 h, but this elevation had no significance. On the other hand, TC, LDL-C, HDL-C, sd-LDL-C, oxidized LDL, apoA-I, apoA-II, apoB, apoC-II, apoC-III, and apoE concentrations were not elevated, which were consistent with our previous findings[11].

We also analyzed several atherogenic indicators and found that the values of non-HDL-C, non-HDL-C/HDL-C, LDL-C/HDL-C, apoB/apoA-I, and non-HDL-C/apoB were not significantly altered, whereas the value of TG/apoB was significantly elevated. Serum concentrations of TG, RLP-C, RLP-TG, TG/apoB and insulin separately analyzed in men and in women (Table 2B and C, respectively), were significantly elevated after, compared with before test meal loading.

The iAUC for TG, RemL-C, RLP-C, RLP-TG, and TG/apoB, which were significantly increased after test meal loading, were significantly greater in men than in women (*p* < 0.05, *p* < 0.005, *p* < 0.005, *p* < 0.01, *p* < 0.05, respectively, Welch’s *t*-test).

**Figure 1:**
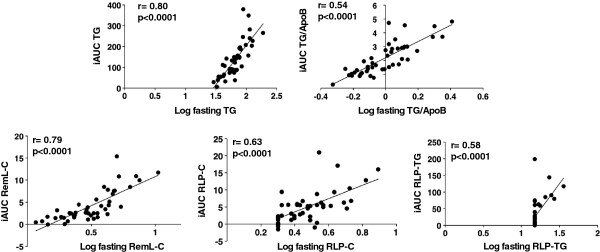
**Correlation between fasting values and the iAUC of TG, RemL-C, RLP-C, RLP-TG, and TG/apoB.** TG, RemL-C, RLP-C, RLP-TG, and TG/apoB were transformed into logarithmic values. Correlations were calculated using the formula for Pearson’s correlation coefficient. Apo, apolipoprotein; RemL-C, remnant lipoprotein cholesterol assayed using “MetaboLead RemL-C”; RLP-C, remnant-like particle-cholesterol assayed using “JIMRO II”; RLP-TG, remnant-like particle-triglyceride; TG, triglycerides.

Fasting serum values and the iAUC of each factor and parameter significantly and positively correlated in all participants (Fig. 1). Fasting serum values for TG, RemL-C, RLP-C, RLP-TG and TG/apoB in men (n = 26) also significantly and positively correlated with the iAUC (r = 0.78, *p* < 0.0001; r = 0.78, *p* < 0.0001; r = 0.60, *p* < 0.005; r = 0.54, *p* < 0.005; r = 0.81, *p* < 0.0001, respectively). Fasting serum values for TG, RemL-C, and TG/apoB in women (n = 19) significantly and positively correlated with the iAUC (r = 0.77, r = 0.76, r = 0.79, respectively; all *p* < 0.0001), but relationships between fasting serum values of RLP-TG and the iAUC could not be estimated because fasting RLP-TG was undetectable in all of the women. Despite the small number of female participants, these results indicated that fasting values of RemL-C, RLP-C, RLP-TG, and TG/apoB in addition to TG are useful markers of postprandial hyperlipidemia in men and women.

### Correlation between fasting adiponectin concentration and fasting lipids and their parameters

There was no significant correlation between adiponectin and several factors (Table 3). As for LPL mass, although we had data from only 21 subjects (men 15, women 6) in the fasting period, a significant correlation with adiponectin was observed in all subjects.

**Table 3:** Correlation between adiponectin and other characteristics

		** Total**		** Men**		** Women**	
		**r**	**p**	**r**	**p**	**r**	**p**
Height	(m)	-0.16	0.31	0.05	0.83	0.24	0.33
Body weight	(kg)	-0.19	0.20	0.01	0.96	-0.01	0.97
Waist circumference	(cm)	-0.26	0.09	-0.08	0.70	-0.21	0.39
BMI	(kg/m^2^)	-0.15	0.33	-0.01	0.95	-0.33	0.17
HbA1c	(%)	-0.04	0.77	0.02	0.94	-0.14	0.58
Hs-CRP	(mg/dL)	-0.21	0.17	-0.07	0.75	-0.15	0.53
LPL mass	(ng/mL)	0.54	0.01	0.34	0.21	0.81	0.05

BMI; body mass index, HbA1c; glycated hemoglobin A1c, Hs-CRP; high-sensitivity C-reactive protein, LPL; lipoprotein lipase. Adiponectin was transformed into logarithmic value. Correlation of adiponectin with other markers was analyzed by Pearson’s correlation coefficient. All factors were derived from 45 subjects (men 26, women 19) except LPL mass (total 21, men 15, women 6).

We examined the correlation between fasting adiponectin concentration and fasting lipids and their parameters (Table 4). Fasting adiponectin concentration positively correlated with HDL-C and apoA-I concentrations, and negatively correlated with values of TG, RemL-C, RLP-C, RLP-TG, non-HDL-C/HDL-C, LDL-C/HDL-C, and TG/apoB. After test meal loading, fasting adiponectin concentration positively correlated with HDL-C and apoA-I concentrations, and negatively correlated with the values of TG, RemL-C, RLP-C, RLP-TG, non-HDL-C/HDL-C, LDL-C/HDL-C, apoB/apoA-I, and TG/apoB from 1 h to 8 h, fully or partially. Thus, fasting adiponectin concentration negatively correlated with postprandial elevation of lipids and their parameters.

**Table 4:** Correlation between adiponectin and parameters concerning lipid and glucose metabolism before and after loading the test meal

		**0**	**1 h**	**2 h**	**4 h**	**6 h**	**8 h**
		***r***	***r***	***r***	***r***	***r***	***r***
TC	(mg/dL)	0.09	0.08	0.06	0.05	0.08	0.06
LDL-C	(mg/dL)	-0.06	-0.06	-0.07	-0.09	-0.08	-0.09
HDL-C	(mg/dL)	0.43*	0.43*	0.45*	0.42*	0.41*	0.41***
Sd-LDL-C	(mg/dL)	-0.11	-0.24	-0.26	-0.26	-0.18	-0.16
OxLDL	(U/mL)	0.03	0.06	0.05	0.08	0.02	0.03
TG	(mg/dL)	-0.42***	-0.39***	-0.40***	-0.48*	-0.40***	-0.42*
Non-HDL-C	(mg/dL)	-0.12	-0.13	-0.15	-0.17	-0.13	-0.15
RemL-C	(mg/dL)	-0.35****	-0.30****	-0.37****	-0.44***	-0.33****	-0.34****
RLP-C	(mg/dL)	-0.38****	-0.36****	-0.39***	-0.45***	-0.28	-0.35****
RLP-TG	(mg/dL)	-0.35**	-0.30****	-0.36****	-0.42*	-0.38****	-0.34****
ApoA-I	(mg/dL)	0.34****	0.37****	0.35****	0.33****	0.34****	0.35****
ApoA-II	(mg/dL)	-0.06	-0.07	-0.11	-0.11	-0.07	-0.07
ApoB	(mg/dL)	-0.09	-0.10	-0.13	-0.13	-0.12	-0.13
ApoC-II	(mg/dL)	-0.29	-0.27	-0.27	-0.32****	-0.27	-0.24
ApoC-III	(mg/dL)	-0.21	-0.18	-0.21	-0.27	-0.20	-0.13
ApoE	(mg/dL)	0.06	0.06	0.03	0.02	0.08	0.10
Non-HDL-C/HDL-C		-0.37****	-0.38***	-0.38***	-0.38***	-0.37****	-0.38***
LDL-C/HDL-C		-0.34****	-0.35****	-0.35****	-0.35****	-0.35****	-0.36****
ApoB/ApoA-I		-0.29	-0.31****	-0.31****	-0.30	-0.31****	-0.32****
Non-HDL-C/ApoB		-0.21	-0.21	-0.14	-0.26	-0.11	-0.14
TG/apoB		-0.40***	-0.39***	-0.37****	-0.43***	-0.33****	-0.35****
Plasma glucose	x(mg/dL)	-0.12	-0.06	-0.04	-0.13	-0.12	-0.11
Insulin	(μU/mL)	-0.17	-0.02	-0.07	-0.22	-0.33****	-0.22

TC; total cholesterol, LDL-C; low-density lipoprotein-cholestrol, HDL-C; high-density lipoprotein-cholesterol, Sd-LDL; small, dense-LDL, OxLDL; oxidized LDL, TG; triglycerides, RemL-C; remnant lipoprotein cholesterol measured with “MetaboLead RemL-C”, RLP-C; remnant-like particle-cholesterol measured with “JIMRO II”, RLP-TG; remnant-like particle-triglycerides, Apo; apolipoprotein. TG, RemL-C, RLP-C, RLP-TG, TG/apoB and adiponectin were transformed into logarithmic values. Correlation of adiponectin with other markers was analyzed by Pearson’s correlation coefficient. *p<0.005, **p<0.001, ***p<0.01, ****p<0.05.

When we examined the relationship between fasting LPL mass and lipids and their parameters before and after test meal loading, there was no significant correlation except some cases of apoC-II at 0, 1, 4, 6, and 8 h and insulin at 6 h (r=−0.44, r=−0.44, r=−0.48, r=−0.47 r=−0.50, r=−0.50, respectively, all p<0.05).

**Figure 2:**
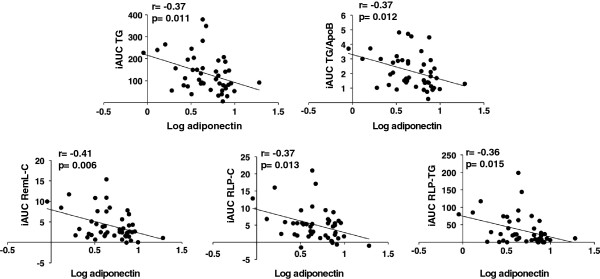
**Correlation between fasting adiponectin concentration and the iAUC of TG, RemL-C, RLP-C, RLP-TG, and TG/apoB.** TG, RemL-C, RLP-C, RLP-TG, TG/apoB, and adiponectin were transformed into logarithmic values. Correlations were calculated using the formula for Pearson’s correlation coefficient. Apo, apolipoprotein; RemL-C, remnant lipoprotein cholesterol assayed using “MetaboLead RemL-C”; RLP-C, remnant-like particle-cholesterol assayed using “JIMRO II”; RLP-TG, remnant-like particle-triglyceride; TG, triglycerides.

We also examined the relationship between adiponectin and iAUC of lipids and their parameters. Fasting adiponectin concentration significantly and negatively correlated with the values of TG, RemL-C, RLP-C, RLP-TG, and TG/apoB, but not with other factors (Fig. 2). When we examined the relationship between LPL and iAUC of them, there was no significant correlation (r=−0.02, p=0.94; r=−0.11, p=0.64; r=−0.24, p=0.30; r=−0.14, p=0.56; r=0.04, p=0.87, respectively).

### Multivariable linear regression analysis for postprandial TG elevation

Because TG strongly correlated with TRL-related values such as RemL-C, RLP-C, RLP-TG, and TG/apoB (r=0.90, r=0.80, r=0.80, r=0.83, p<0.0001, respectively), we constructed the model of multivariable linear regression analysis without fasting TG and adjusted by sex, BMI, age, and waist circumference as we described in Methods. RemL-C, RLP-C, RLP-TG, and TG/apoB were significant factors, but adiponectin was not (Table 5). Especially, adjusted R-squared of RemL-C and TG/apoB were 0.63 and 0.55, respectively. Considering the correlation between fasting adiponectin and fasting lipids, we performed multivariable linear regression analysis without lipids and their parameters. However, we could not uncover a significant relationship between fasting adiponectin concentrations and postprandial TG elevation (*p* = 0.09).

**Table 5:** Multivariable linear regression analysis for postprandial TG elevation (adjusted by sex, BMI, age, and waist circumference)

	**β Coefficient**	**SE**	**95 % CI**	***p***	**Standardized partial**	**Adjusted**
					**regression coefficient**	**R-squared**
RemL-C	0.67	0.09	0.48 to 0.86	<0.01	0.72	0.63
RLP-C	0.71	0.17	0.37 to 1.06	<0.01	0.55	0.41
RLP-TG	1.21	0.36	0.48 to 1.93	<0.01	0.48	0.33
TG/apoB	0.81	0.13	0.54 to 1.08	<0.01	0.66	0.55
Adiponectin	-0.21	0.12	-0.45 to 0.03	0.09	-0.27	0.20

## Discussion

The present study demonstrated that fasting TRL-related values of TG, RemL-C, RLP-C, RLP-TG, and TG/apoB were useful tools for predicting postprandial hyperlipidemia and that fasting adiponectin concentrations correlated with the fasting values of these lipids and parameters in young healthy individuals, although adiponectin was not a significant predictor in multivariable linear regression analysis.

Many studies have examined the relationship between adiponectin and dyslipidemia. Baratta et al. reported that the relationship between adiponectin and fasting lipid values is independent of body fat mass [21]. Kazumi et al. and Heliövaara et al. demonstrated that hypoadiponectinemia is more closely related to adiposity and fasting dyslipidemia than insulin resistance in young healthy men [22,23]. Interestingly, Yoshida et al. demonstrated the lower adiponectin concentrations in type IIb hyperlipidemia compared to normolipidemia, type IV, and type IIa hyperlipidemia in type II diabetes patients [24]. On the other hand, the relationship between adiponectin and postprandial hyperlipidemia has not been investigated in detail. Rubin et al. demonstrated that postprandial plasma adiponectin values decreased after oral loading with 75 g of glucose or with a high-fat meal (58 g of fat) and that postprandial TG concentrations correlated with fasting adiponectin concentration in a 45- to 65-year-old population including those with metabolic syndrome [16]. Moreover, Maruyama et al. found lower TG and RLP-TG concentrations among individuals with type 1 diabetes and a high, rather than a low baseline HMW adiponectin concentration at baseline and at 3 h after test meal loading [17]. The present study demonstrated that fasting adiponectin concentration positively correlated with fasting HDL-C and apoA-I concentrations, and negatively correlated with fasting and postprandial values of TG, RemL-C, RLP-C, RLP-TG, non-HDL-C/HDL-C, LDL-C/HDL-C and TG/apoB. These are new findings compared with our previous study [11]. However, multivariable linear regression analysis showed that adiponectin was not significant for predicting postprandial hyperlipidemia, which is difficult to explain. Adiponectin participates in the metabolism of the visceral fat mass, glucose and lipids, but it might not reflect dynamic changes such as lipid concentrations that elevate after test meal loading. In addition, the small number of samples derived from only healthy subjects might have affected this finding. Patients with hyperlipidemia, diabetes or metabolic syndrome often have delayed TRL clearance [10]. Although we may find other results if we include older subjects or these patients, it is also necessary to elucidate the significant predictive factors in young healthy subjects, because our purpose of the present study is to elucidate the significant predictors of postprandial TG elevation. On the other hand, since adiponectin concentrations are considerably higher in women than in men, the significance of adiponectin needs to be separately analyzed in men and women.

A definition or standard method other than TG elevation has not been established for predicting postprandial hyperlipidemia. This complicates resolving which factor among those associated with postprandial hyperlipidemia is the most useful for predicting CVD. Some studies have demonstrated the superiority of non-fasting, over fasting TG concentrations for predicting CVD [6,7]. Oka et al. notably demonstrated that waist circumference is more closely related to postprandial, than to fasting TG [25]. Moreover, the lipid profile in metabolic syndrome includes elevated TG and remnant lipoproteins, decreased LDL particle size and low HDL-C concentrations [10]. Thus, not only fasting TG values, but also other parameters might be required to assess CVD risk. Remnant lipoproteins play an important role in atherogenesis and their concentrations are useful to understand metabolic disorders and to predict CVD [10,26,27]. Ai et al. reported that RLP-C and RLP-TG, but not the TG response to an oral fat load are significantly increased in hyperinsulinemic patients with type 2 diabetes [28]. We also found that fasting serum concentrations of remnant lipoproteins might be useful to detect postprandial hyperlipidemia even in normolipidemic individuals [11]. Here, we confirmed that remnant lipoproteins during the fasting period can predict postprandial hyperlipidemia. The purpose of our study was to elucidate the factors that predict postprandial hyperlipidemia besides TG, because it is difficult to select high-risk patients with TG alone. However, TG strongly correlated with TRL-related values including RemL-C, RLP-C, RLP-TG, and TG/apoB, so we constructed the regression model without fasting TG. Multivariable linear regression analysis identified RemL-C, RLP-C, RLP-TG, and TG/apoB as significant predictors of postprandial TG elevation. Taking these findings together, these TRL-related values might be clinically useful for predicting postprandial hyperlipidemia.

Another novel outcome of the present study is that the amount of postprandial lipid elevation differs between men and women. Fasting values of TG, RemL-C, RLP-C, RLP-TG, non-HDL-C/HDL-C, LDL-C/HDL-C, and apoB/apoA-I were significantly greater in men than in women (Table 2B and C). Furthermore, the iAUCs of TG, RemL-C, RLP-C, RLP-TG, and TG/apoB were also significantly greater in men than in women. These results suggest that men are more susceptible to postprandial hyperlipidemia. The difference in lipoprotein metabolism between men and women may be caused by several mechanisms including lipoprotein lipase and lecithin-cholesterol acyltransferase activities (LCAT) activities, and gender specific hormonal effects [8-10,29]. Higher adiponectin concentrations in women may also influence TRL metabolism (Table 1) [14,15,21-24]. Although we did not check the activities of enzymes and menstrual cycle of each woman in the present study, these differences in fasting and postprandial TRL-related lipid concentrations between men and women should be considered when distinguishing and predicting individuals at high-risk for postprandial hyperlipidemia.

We measured the serum concentrations of several lipid markers. Postprandial accumulation of TRL was strongly associated with the increased prevalence of sd-LDL in patients with myocardial infarction [30]. However, sd-LDL concentrations were decreased in the present study of young healthy individuals. Ogita et al. demonstrated that serum sd-LDL concentrations decrease after meals, increase during the night and peak just before breakfast [31]. Hirayama et al. also demonstrated a similar decrease in the sd-LDL concentrations after breakfast; they speculated that sd-LDL permeates the vascular walls more easily, and might be more susceptible than buoyant LDL to entrapment in vascular subendothelial spaces [32]. The precise mechanism should be addressed in future studies. Regardless, postprandial changes in sd-LDL might differ between healthy individuals and patients with CVD.

We also found that the oxidized LDL concentration did not significantly change. Although oxidized LDL in patients with CAD is postprandially elevated [33], concentrations in healthy individuals have not been investigated in detail. One study has found that oxidized LDL concentrations are not elevated in individuals with normal glucose tolerance during oral glucose tolerance test [34]. Postprandial changes in oxidized LDL concentrations should be examined and compared with those of patients with CAD.

Non HDL-C is an excellent predictor of atherosclerotic risk [35,36] and it is free of dietary variations [36,37]. Ogita et al. also demonstrated that serum concentrations of TC, HDL-C, and LDL-C do not change remarkably in healthy individuals [31]. The present study also did not identify significant changes in TC, HDL-C, LDL-C and non HDL-C concentrations among young healthy individuals. Some studies have demonstrated that LDL-C and HDL-C concentrations decrease during the day in a rhythmic circadian manner [38,39]. We also found decreased LDL-C and HDL-C concentrations, but the changes were not statistically significant. Thus, TC and non HDL-C concentrations were not significantly altered, although the postprandial value of remnant cholesterol was increased.

This study has some limitations. Although others have associated adiponectin concentrations with body weight, waist circumference, BMI, HbA1c and hs-CRP [40,41], we found no significant correlations because we measured these values in young individuals without metabolic syndrome or diabetes.

The RemL-C concentrations increased after test meal loading, albeit without significance, which is consistent with previous findings [11]. Lipoproteins targeted by both RLP-C and RemL-C include remnants of both chylomicrons and VLDL [10]. Concentrations of RemL-C and RLP-C closely correlate in patients with coronary artery disease, but the sensitivity of detecting chylomicron remnant (exogenous) and VLDL remnant (endogenous) lipoproteins might differ between analytical methods [10]. By detailed analysis with the high performance liquid chromatography method, Yoshida et al. demonstrated that they found higher concentrations of chylomicron cholesterol in serum samples with RemL-C < RLP-C, but high concentrations of intermediate-density lipoprotein (IDL)-cholesterol (VLDL remnant cholesterol) in samples with RemL-C > RLP-C [42]. Similarly, we and others also reported that methods for measuring RLP-C and RLP-TG might be more sensitive to chylomicron remnant-cholesterol and -triglycerides, whereas those for RemL-C might be more suitable for IDL-cholesterol [11,20,43]. This may cause a difference in elevation after test meal loading and in multivariable linear regression analysis between RemL-C and RLP-C in the present study.

We did not evaluate the effect of activities such as aerobic exercise that can decrease TG and remnant concentrations and increase adiponectin concentrations [44]. Factors that can predict postprandial hyperlipidemia should be investigated in larger populations including individuals with metabolic syndrome, diabetes and CVD.

## Conclusion

Fasting TRL-related values, especially RemL-C, RLP-C, RLP-TG, and TG/apoB are useful predictors of postprandial hyperlipidemia in young healthy individuals. Although the fasting adiponectin concentration correlates with the fasting values of TG, RemL-C, RLP-C, RLP-TG and TG/apoB, it is not a significant predictor of postprandial hyperlipidemia in multivariable linear regression analysis.

## Abbreviations

TG: Triglycerides; CVD: Cardiovascular diseases; TRL: Triglyceride-rich lipoproteins; VLDL: Very-low-density lipoprotein; apo: Apolipoprotein; HMW: High molecular weight; RLP-C: Remnant-like particle-cholesterol; RLP-TG: Remnant-like particle-triglycerides; RemL-C: Remnant lipoprotein cholesterol; BMI: Body mass index; LDL-C: LOW-density lipoprotein-cholesterol; HDL-C: High-density lipoprotein-cholesterol; sd-LDL-C: Small dense LDL-cholesterol; hsCRP: High-sensitivity C-reactive protein; HbA1c: Glycosylated hemoglobin A1c; iAUC: Incremental area under the curve; OxLDL: Oxidized LDL; SE: Standard error; CI: Confidence interval.

## Competing interests

There is no conflict of interest for this paper.

## Authors’ contributions

All authors should have made substantial contributions to all of the following: (1) the conception and design of the study, or acquisition of data, or analysis and interpretation of data, (2) drafting the article or revising it critically for important intellectual content, (3) final approval of the version to be submitted. Especially, TN, ST, HO, IS, MY, HK, SK, and YF actually performed the examination according to the protocol. TN, SY, YI and YF were involved in protocol development, gaining ethical approval, and patient recruitment. TN, DS, TK and YF were involved in data analysis.

## Declarations

Submission of our article implies that the work described has not been published previously that it is not under consideration for publication elsewhere, that its publication is approved by all authors and tacitly or explicitly by the responsible authorities where the work was carried out, and that, if accepted, it will not be published elsewhere in the same form, in English or in any other language, including electronically without the written consent of the copyright-holder.
