# Correction: fasting remnant lipoproteins can predict postprandial hyperlipidemia

**DOI:** 10.1186/1476-511X-13-68

**Published:** 2014-04-17

**Authors:** Tomoki Nagata, Daisuke Sugiyama, Takako Kise, Satomi Tsuji, Hideo Ohira, Itsuko Sato, Mari Yamamoto, Hitomi Kohsaka, Seiji Kawano, Shizuya Yamashita, Yuichi Ishikawa, Yoshio Fujioka

**Affiliations:** 1 Division of Clinical Nutrition, Faculty of Nutrition, Kobe Gakuin University, 518 Arise, Ikawadani-cho, Nishi-ku, Kobe 651-2180, Japan; 2 Section of Evidence-based Laboratory Medicine, Division of Clinical Pathology and Immunology, Department of Internal Medicine Related, Kobe University Graduate School of Medicine, Kobe, Japan; 3 Department of Clinical Laboratory, Kobe University Hospital, Kobe, Japan; 4 Department of Cardiovascular Medicine, Osaka University Graduate School of Medicine, Osaka, Japan; 5 Department of Internal Medicine, Kakogawa West City Hospital, Kakogawa, Japan; 6 Division of Clinical Laboratory, Shimane Prefectural Central Hospital, Shimane, Japan; 7 Department of Preventive Medicine and Public Health, School of Medicine, Keio University, Tokyo, Japan; 8 Division of Nutrition Guidance, Kakogawa East City Hospital, Kakogawa, Japan

## Correction

After publication of this article [1], we noted formatting errors in Table 2. Elapsed time (h) columns at lines 25 to 43 were out of alignment and the true 8 h data were missing. The corrected version of Table 2 is below (Table 1).

**Table 1:** Lipid and glucose parameters for before and 1 – 8 h after loading with test meal

**A. All participants**							
Elapsed time (h)							
		0	1	2	4	6	8
TC	(mg/dL)	182.4 ± 32.8	180.2 ± 32.8	178.2 ± 31.3	180.4 ± 30.1	184.0 ± 31.6	188.2 ± 32.5
LDL-C	(mg/dL)	98.5 ± 26.1	96.3 ± 25.5	95.6 ± 25.5	96.7 ± 24.7	99.8 ± 25.6	102.1 ± 26.2
HDL-C	(mg/dL)	70.1 ± 15.4	68.3 ± 15.4	66.2 ± 14.1	68.3 ± 15.0	70.2 ± 15.7	72.0 ± 16.0
Sd-LDL-C	(mg/dL)	22.4 ± 12.1	19.8 ± 8.8	18.8 ± 8.5	18.6 ± 7.1	18.6 ± 7.1	19.0 ± 7.0
OxLDL	(U/mL)	7.38 ± 4.96	7.04 ± 4.98	7.25 ± 5.74	7.41 ± 5.53	7.71 ± 5.41	8.38 ± 5.46
TG	(mg/dL)	69.8 ± 31.2	92.1 ± 41.0*	103.9 ± 51.4^†^	72.9 ± 40.4	70.7 ± 34.3	53.9 ± 23.8
Non-HDL-C	(mg/dL)	112.3 ± 30.1	112.0 ± 29.9	112.0 ± 29.3	112.0 ± 28.4	113.8 ± 28.7	116.3 ± 29.4
RemL-C	(mg/dL)	3.51 ± 1.84	4.35 ± 2.11	4.29 ± 2.46	3.90 ± 2.38	3.22 ± 1.61	2.98 ± 1.40
RLP-C	(mg/dL)	3.20 ± 1.30	4.22 ± 1.91*	5.01 ± 2.42^‡^	3.88 ± 1.94	3.03 ± 1.02	2.82 ± 0.88
RLP-TG	(mg/dL)	16.3 ± 4.0	25.5 ± 14.4^†^	32.6 ± 21.8^‡^	21.1 ± 12.3	15.8 ± 3.4	15.3 ± 2.0
ApoA-I	(mg/dL)	161.2 ± 26.6	159.8 ± 27.3	158.7 ± 25.2	160.3 ± 25.2	163.3 ± 26.4	165.7 ± 27.0
ApoA-II	(mg/dL)	37.6 ± 6.0	37.3 ± 6.2	36.8 ± 6.0	37.4 ± 5.9	37.7 ± 5.9	38.4 ± 6.1
ApoB	(mg/dL)	66.6 ± 15.3	65.7 ± 15.1	65.0 ± 14.5	66.2 ± 14.5	67.9 ± 14.8	69.3 ± 15.2
ApoC-II	(mg/dL)	3.2 ± 1.2	3.3 ± 1.2	3.3 ± 1.2	3.3 ± 1.2	3.3 ± 1.1	3.3 ± 1.1
ApoC-III	(mg/dL)	9.5 ± 2.4	9.8 ± 2.5	9.4 ± 2.3	9.2 ± 2.2	9.0 ± 2.1	9.0 ± 2.2
ApoE	(mg/dL)	4.3 ± 1.1	4.3 ± 1.1	4.3 ± 1.0	4.2 ± 1.1	4.1 ± 1.0	4.2 ± 1.1
Non-HDL-C/HDL-C		1.70 ± 0.73	1.75 ± 0.75	1.80 ± 0.75	1.75 ± 0.76	1.73 ± 0.74	1.72 ± 0.73
LDL-C/HDL-C		1.50 ± 0.60	1.50 ± 0.61	1.52 ± 0.61	1.50 ± 0.61	1.51 ± 0.60	1.50 ± 0.60
ApoB/apoA-I		0.43 ± 0.13	0.42 ± 0.13	0.42 ± 0.13	0.43 ± 0.13	0.43 ± 0.13	0.43 ± 0.13
Non-HDL-C/apo B		1.67 ± 0.10	1.69 ± 0.10	1.71 ± 0.11	1.68 ± 0.10	1.67 ± 0.10	1.67 ± 0.10
TG/apoB		1.06 ± 0.44	1.41 ± 0.56^§^	1.61 ± 0.71^‡^	1.25 ± 0.61	0.93 ± 0.40	0.79 ± 0.32
Plasma glucose	(mg/dL)	89.9 ± 5.7	96.9 ± 20.0^§^	87.5 ± 10.8	87.6 ± 4.9	88.1 ± 5.7	87.2 ± 5.7
Insulin	(μU/mL)	5.6 ± 2.4	42.4 ± 24.6^‡^	18.5 ± 14.1^‡^	5.0 ± 2.2	4.2 ± 2.1	3.8 ± 1.7
**B. Men**							
TC	(mg/dL)	183.1 ± 33.8	181.4 ± 34.5	180.0 ± 33.3	182.4 ± 32.5	185.0 ± 33.2	189.5 ± 34.9
LDL-C	(mg/dL)	101.1 ± 28.2	99.0 ± 27.5	99.3 ± 27.7	99.8 ± 26.9	102.7 ± 27.8	105.3 ± 28.8
HDL-C	(mg/dL)	66.3 ± 16.7	64.7 ± 17.4	62.5 ± 15.5	65.1 ± 17.2	66.5 ± 17.4	68.3 ± 17.9
Sd-LDL-C	(mg/dL)	23.9 ± 11.0	22.2 ± 10.2	21.4 ± 9.5	20.4 ± 9.0	19.8 ± 8.0	20.6 ± 7.8
OxLDL	(U/mL)	7.98 ± 5.48	7.91 ± 5.54	8.12 ± 6.19	8.32 ± 6.11	8.70 ± 6.22	9.44 ± 6.12
TG	(mg/dL)	78.9 ± 36.0	106.3 ± 45.1	119.5 ± 58.9^†^	94.8 ± 51.2	68.6 ± 33.7	60.2 ± 28.6
Non-HDL-C	(mg/dL)	116.8 ± 32.5	116.8 ± 32.2	117.5 ± 31.8	117.3 ± 31.1	118.5 ± 31.2	121.2 ± 32.5
RemL-C	(mg/dL)	4.08 ± 2.13	4.99 ± 2.42	5.16 ± 2.81	4.69 ± 2.80	3.67 ± 1.91	3.36 ± 1.66
RLP-C	(mg/dL)	3.62 ± 1.51	5.00 ± 2.03*	5.82 ± 2.76^‡^	4.53 ± 2.23	3.31 ± 1.13	3.04 ± 1.01
RLP-TG	(mg/dL)	17.3 ± 5.1	30.0 ± 16.7^§^	37.8 ± 26.2^‡^	24.7 ± 15.2	16.4 ± 4.5	15.6 ± 2.6
ApoA-I	(mg/dL)	155.7 ± 29.2	154.4 ± 29.9	154.3 ± 28.7	155.2 ± 29.0	157.9 ± 29.4	160.6 ± 30.4
ApoA-II	(mg/dL)	33.8 ± 6.7	38.7 ± 6.8	38.4 ± 6.6	38.8 ± 6.5	39.1 ± 6.7	39.8 ± 6.8
ApoB	(mg/dL)	68.8 ± 16.3	68.3 ± 16.3	67.5 ± 15.6	68.8 ± 15.8	70.5 ± 16.0	71.8 ± 16.7
ApoC-II	(mg/dL)	3.5 ± 1.1	3.6 ± 1.1	3.6 ± 1.2	3.6 ± 1.2	3.6 ± 1.1	3.5 ± 1.1
ApoC-III	(mg/dL)	9.7 ± 2.6	10.2 ± 2.8	9.8 ± 2.7	9.5 ± 2.5	9.2 ± 2.3	9.2 ± 2.4
ApoE	(mg/dL)	4.1 ± 1.1	4.1 ± 1.1	4.2 ± 1.0	4.0 ± 1.1	3.9 ± 1.0	4.0 ± 1.0
Non-HDL-C/HDL-C		1.90 ± 0.85	1.96 ± 0.88	2.02 ± 0.87	1.96 ± 0.89	1.94 ± 0.86	1.92 ± 0.86
LDL-C/HDL-C		1.64 ± 0.71	1.66 ± 0.72	1.70 ± 0.70	1.66 ± 0.72	1.67 ± 0.71	1.66 ± 0.71
ApoB/apoA-I		0.46 ± 0.15	0.46 ± 0.15	0.46 ± 0.15	0.46 ± 0.16	0.46 ± 0.15	0.46 ± 0.15
Non-HDL-C/apo B		1.69 ± 0.10	1.70 ± 0.11	1.73 ± 0.12	1.70 ± 0.11	1.67 ± 0.10	1.68 ± 0.11
TG/apoB		1.17 ± 0.52	1.58 ± 0.63	1.79 ± 0.84^†^	1.41 ± 0.73	1.00 ± 0.49	0.86 ± 0.39
Plasma glucose	(mg/dL)	91.1 ± 5.9	97.7 ± 19.9	85.7 ± 9.7	89.2 ± 4.6	88.7 ± 5.9	87.7 ± 5.9
Insulin	(μU/mL)	5.0 ± 2.1	33.6 ± 13.7^‡^	11.6 ± 7.4^†^	4.5 ± 2.0	3.8 ± 2.3	3.7 ± 1.8
**C. Women**							
TC	(mg/dL)	181.4 ± 32.3	178.6 ± 31.3	175.7 ± 29.1	177.6 ± 27.1	182.7 ± 30.0	186.5 ± 29.6
LDL-C	(mg/dL)	95.1 ± 23.3	92.6 ± 22.6	90.6 ± 21.8	92.5 ± 21.4	95.7 ± 22.2	97.7 ± 22.1
HDL-C	(mg/dL)	75.3 ± 12.0	73.2 ± 10.9	71.3 ± 10.3	72.8 ± 10.4	75.4 ± 11.6	77.0 ± 11.6
Sd-LDL-C	(mg/dL)	20.3 ± 13.6	16.5 ± 4.9	15.3 ± 5.3	16.1 ± 4.6	16.8 ± 5.5	16.7 ± 5.2
OxLDL	(U/mL)	6.55 ± 4.15	5.86 ± 3.91	6.06 ± 4.99	6.17 ± 4.48	6.37 ± 3.83	6.92 ± 4.10
TG	(mg/dL)	57.3 ± 16.8	72.7 ± 24.6	82.6 ± 28.3^||^	63.7 ± 17.0	52.8 ± 12.4	45.2 ± 10.3
Non-HDL-C	(mg/dL)	106.1 ± 25.9	105.4 ± 25.8	104.4 ± 24.2	104.8 ± 23.2	107.3 ± 24.3	109.5 ± 23.9
RemL-C	(mg/dL)	2.73 ± 0.95	3.47 ± 1.16	3.11 ± 1.13	2.83 ± 0.95	2.60 ± 0.77	2.45 ± 0.69
RLP-C	(mg/dL)	2.62 ± 0.60	3.16 ± 1.06	3.90 ± 1.22^‡^	2.98 ± 0.88	2.66 ± 0.72	2.52 ± 0.54
RLP-TG	(mg/dL)	15	19.4 ± 7.0	25.5 ± 11.0^‡^	16.2 ± 2.1	15.0 ± 0.2	15
ApoA-I	(mg/dL)	168.7 ± 21.0	167.3 ± 21.8	164.8 ± 18.5	167.3 ± 17.3	170.7 ± 20.1	172.7 ± 20.3
ApoA-II	(mg/dL)	36.0 ± 4.6	35.4 ± 4.7	34.6 ± 4.4	35.3 ± 4.3	35.8 ± 4.1	36.5 ± 4.5
ApoB	(mg/dL)	63.7 ± 13.6	62.2 ± 12.9	61.5 ± 12.5	62.6 ± 12.2	64.4 ± 12.6	65.8 ± 12.4
ApoC-II	(mg/dL)	2.8 ± 1.1	2.9 ± 1.1	2.9 ± 1.1	2.9 ± 1.0	2.9 ± 1.0	2.9 ± 1.1
ApoC-III	(mg/dL)	9.1 ± 2.0	9.3 ± 2.0	8.9 ± 1.7	8.8 ± 1.6	8.6 ± 1.8	8.7 ± 1.8
ApoE	(mg/dL)	4.7 ± 1.0	4.6 ± 1.1	4.5 ± 1.1	4.5 ± 1.1	4.5 ± 1.1	4.6 ± 1.1
Non-HDL-C/HDL-C		1.43 ± 0.38	1.46 ± 0.40	1.48 ± 0.39	1.47 ± 0.40	1.45 ± 0.39	1.45 ± 0.37
LDL-C/HDL-C		1.28 ± 0.33	1.28 ± 0.33	1.29 ± 0.33	1.29 ± 0.34	1.29 ± 0.33	1.29 ± 0.32
ApoB/apoA-I		0.38 ± 0.08	0.37 ± 0.08	0.38 ± 0.08	0.38 ± 0.08	0.38 ± 0.08	0.38 ± 0.08
Non-HDL-C/apo B		1.66 ± 0.11	1.68 ± 0.09	1.69 ± 0.11	1.67 ± 0.08	1.66 ± 0.10	1.66 ± 0.10
TG/apoB		0.92 ± 0.26	1.17 ± 0.32^‡^	1.36 ± 0.40^†^	1.04 ± 0.29	0.84 ± 0.22	0.70 ± 0.17
Plasma glucose	(mg/dL)	88.3 ± 5.2	95.8 ± 20.6	89.9 ± 12.1	85.5 ± 5.0	87.1 ± 5.1	86.5 ± 5.7
Insulin	(μU/mL)	6.5 ± 2.6	54.1 ± 31.1^‡^	27.9 ± 15.7^‡^	5.6 ± 2.4	4.8 ± 1.8	3.9 ± 1.5

*Apo* apolipoprotein; *HDL-C* high-density lipoprotein cholesterol; *LDL-C* low-density lipoprotein cholesterol; *OxLDL* oxidized *LDL*; *RemL-C* remnant lipoprotein cholesterol measured using “MetaboLead RemL-C”; *RLP-C* remnant-like particle-cholesterol measured with “JIMRO II”; *RLP-TG* remnant-like particle-triglycerides; *Sd-LDL-C* small, dense-LDL cholesterol; *TC* total cholesterol; *TG* triglycerides. TG, RemL-C, RLP-C, RLP-TG, and TG/apoB were transformed into logarithmic values. Values are expressed as means ± SD. *p < 0.05, ^†^*p* < 0.005, ^‡^*p* < 0.0001, ^§^*p* < 0.01, ^||^*p* < 0.001, vs. time 0 (repeated ANOVA with Dunnett’s test).
